# Improving Gut Microbiota and Growth Performance of Edible Crickets (*Gryllus bimaculatus*) by the Probiotic *Lactiplantibacillus plantarum* TPL-2 from the Guts of the Termite, *Termes propinquus*

**DOI:** 10.3390/microorganisms14030660

**Published:** 2026-03-14

**Authors:** Kittipong Chanworawit, Putsawee Tomtong, Pachara Wangsoonthorn, Kiattawee Choowongkomon, Pinsurang Deevong

**Affiliations:** 1 Department of Microbiology, Faculty of Science, Kasetsart University, Bangkok 10900, Thailand; 2 Department of Biochemistry, Faculty of Science, Kasetsart University, Bangkok 10900, Thailand

**Keywords:** probiotics, lactic acid bacteria, *Lactiplantibacillus plantarum*, termite gut, edible crickets, *Gryllus bimaculatus*, gut microbiota

## Abstract

Termite guts represent a unique microbial habitat harboring bacteria with potential probiotic properties, owing to their ability to inhibit pathogenic microorganisms. This study investigated the probiotic characteristics of lactic acid bacteria newly isolated from the guts of the termite *Termes propinquus*, aiming to enhance growth performance and reduce the incidence of foodborne pathogen contamination in the commonly consumed edible two-spotted crickets (*Gryllus bimaculatus*). In this study, five morphologically different bacteria (TPL-1 to TPL-5) were isolated and respectively identified as *Levilactobacillus brevis*, *Lactiplantibacillus plantarum*, *Streptococcus anginosus*, *Companilactobacillus alimentarius*, and *Aerococcus viridans* based on 16S rRNA gene sequences and MALDI-TOF MS. All isolates were evaluated for tolerance to stressful conditions (pH 2.5 and 0.3% bile salts), cell surface properties, antioxidant activity, antimicrobial activity against foodborne pathogens, safety profiles, and adhesion to human colon adenocarcinoma cells (Caco-2 and HT-29). Among them, *Lactiplantibacillus plantarum* TPL-2 demonstrated the strongest probiotic attributes and was further assessed for anti-adhesion activity against foodborne pathogens and *in vivo* effects on the crickets. Dietary supplementation with *Lb. plantarum* TPL-2 significantly improved cricket growth, survival, and gut microbiota homeostasis. These findings point to the prospect of termite-derived lactic acid bacteria as beneficial probiotics for use in biotechnological applications and edible insect farming.

## 1. Introduction

Probiotics are beneficial microorganisms, primarily bacteria in the *Bacillus* group, including the genera *Bacillus* and *Paenibacillus* and the lactic acid bacteria (LAB) group, such as *Bifidobacterium*, *Pediococcus*, *Streptococcus*, *Lactococcus*, *Enterococcus*, and *Lactobacillus* (presently reclassified into the genera *Lacticaseibacillus*, *Lactiplantibacillus*, *Levilactobacillus*, and *Companilactobacillus*), that are found in fermented foods, animal gastrointestinal tracts, and natural environments, and, when consumed in adequate amounts, provide health benefits to the host [1,2,3]. Insect probiotics are live microorganisms that are supplemented to the diet of insects to improve their health, growth performance, and resistance to diseases and help establish gut microbiome modulation in them [4]. Lactic acid bacteria with probiotic properties are found in insect gastrointestinal tracts. Many lactic acid bacteria have been isolated from insects, and their ability to promote growth and survival and prevent pathogen invasion in insects, including the bean bug (*Riptortus pedestris*) [5], mealworm (*Tenebrio molitor*) [6], red flour beetle (*Tribolium castaneum*) [7], and Mediterranean fruit fly (*Ceratitis capitata*) [8], has been reported.

Insects are the most diverse group of animals on earth, with millions of species in various environments, and a commonly consumed food source offering a sustainable and nutrient-rich alternative to traditional livestock [9]. The microbial community in insect intestinal systems plays a crucial role in various aspects of insects’ health, including supporting digestion, development of immunity, and improved nutrition [10]. In Southeast Asian countries, edible insects like crickets, silkworms, and grasshoppers are common street food as a healthy and delicious snack. They are recognized as an alternate source of protein and other valuable nutrients, usable in various dietary applications. The two-spotted cricket, *Gryllus bimaculatus* (Orthoptera: Gryllidae), is a widely cultivated edible species authorized for human consumption in Thailand [11,12]. The edible cricket species are known for their rich protein content, often exceeding 60–70% on a dry weight basis, and contain essential amino acids that humans need, making them a complete protein source [13]. However, foodborne pathogens, such as *Salmonella* spp., *Staphylococcus aureus*, *Pseudomonas* spp., *Escherichia coli*, *Listeria monocytogenes*, and endospore-forming bacteria harbored by insects have been reported to induce diseases in insects and can be transmitted to humans [14,15]. The pathogen contamination may arise through multiple routes, including the insect’s habitat, and the quality of the feed used in insect farming might introduce diseases [16]. According to Kikuchi and Environments [17], the metagenomic analysis of microbes in edible insects poses a negligible risk to consumer safety. One alternative way to reduce the pathogen number in insects involves using microbial control agents such as probiotics. Moreover, supplementing insect feed with probiotics can be used in insect farming to improve insect growth rates and health and potentially protect the insects, farmers, and consumers from pathogens and their microbial toxins [18,19].

Termites (Isoptera) are edible insects and a source of protein for many cultures worldwide, particularly in Africa, Asia, and South America [20,21]. They are a beneficial source of protein, fat, and certain vitamins and minerals and are considered safe for human consumption when cooked properly. Termite guts have unique digestive systems teeming with diverse microorganisms, including bacteria, archaea, and protozoa, which are essential for the digestion of cellulose and other complex carbohydrates found in wood and soil materials. Termite gut bacteria are also capable of producing antimicrobial compounds that are considered target-specific against invasive species [22]. Tomtong and Deevong (2024) [21] have reported the isolation and evaluation of numerous probiotic *Bacillus* species from the guts of *Termes propinquus*. However, the study of lactic acid bacteria as a probiotic isolated from the guts of the termite *T. propinquus* is still limited. Thus, the present study aims to isolate the probiotic lactic acid bacteria from termite guts that can be used as microbial agents to promote the growth, quality, and safety of the edible crickets (two-spotted cricket, *G. bimaculatus*) by enhancing the balance of gut microbiota, reducing pathogen contamination, and protecting against infections that can hinder growth. This study provides probiotic knowledge in food microbiology and biotechnology.

## 2. Materials and Methods

### 2.1. Termites, Crickets, and Ethical Approval

The soil/wood-feeding higher termites (*T. propinquus*) were collected from the Sakaerat Environmental Research Station, Nakhon Ratchasima province, Thailand (14°30′34.3″ N 101°55′50.9″ E), and 14-day-old two-spotted crickets (*G. bimaculatus*; calculated from the date of hatching) were obtained from a cricket farm adhering to the Good Agricultural Practices (GAP) standards in Thailand. The protocol was approved and permitted by the KASETSART UNIVERSITY Institutional Animal Care and Use Committee (Approval ID. ACKU68-SCI-009 and ACKU68-SCI-010) and found to be in accordance to the guidelines of animal care and use under the Ethical Review Board of the Office of National Research Council of Thailand (NRCT) for the conduction of the scientific research.

### 2.2. Isolation and Identification of Lactic Acid Bacteria from the Guts of Termites

Termite samples were transported to the laboratory for isolation within 12 h. The termite surface was washed three times with sterile distilled water on an ice-cold plate. Overall, 30 guts of *T. propinquus* were squeezed and homogenized with a sterile pestle and mixed by vortexing. The gut homogenate was serially diluted in 0.85% NaCl and spread on De Man–Rogosa–Sharpe (MRS) agar, pH 6.5 (HiMedia Laboratories Pvt. Ltd., Mumbai, India). After incubation at 37 °C for 24 h in low-oxic conditions using the candle jar method, bacterial colonies were picked and cross-streaked on MRS agar. Morphologically different colonies of lactic acid bacteria were selected for the catalase production test using 3% (*v*/*v*) H_2_O_2_ and Gram’s staining. Moreover, bacterial genomic DNA of the selected isolates was extracted using the Genomic DNA Purification Kit (Thermo Fisher Scientific, Waltham, MA, USA) according to the manufacturer’s instructions. The 16S rRNA gene (~1.5 kb) was amplified by polymerase chain reaction (PCR) using the 16S rRNA universal primer pair: forward 616V (5′-AGAGTTGATYMTGGCTC-3′) and reverse 1492R (5′-GGYTACCTTGTTACGACTT-3′) [23], according to the method of Loy et al. [24]. The PCR reaction was performed by 2× DreamTaq Green PCR Master Mix (Thermo Fisher Scientific, Waltham, MA, USA). The DNA sequencing of PCR products was conducted by Macrogen, Inc. (Seoul, Republic of Korea). The nucleotide sequences were analyzed using BioEdit and taxonomically identified by the BLAST program (https://blast.ncbi.nlm.nih.gov/Blast.cgi, accessed on 29 July 2025). Reference sequences of closely related species were obtained from the NCBI GenBank database based on BLAST results. Representative strains with high sequence similarity and coverage were selected to construct the phylogenetic tree and ensure accurate taxonomic placement of the isolate. The phylogenetic tree was constructed using Molecular Evolutionary Genetics Analysis Version 11 (MEGA11) after multiple sequence alignments by ClustalW, based on the maximum-likelihood method. In addition to the molecular identification, the Matrix Assisted Laser Desorption Ionization Time-of-Flight (MALDI-TOF) mass spectrometry identification was generated by the VITEK^®^MS system (version 3.2; BioMerieux, Durham, NC, USA).

### 2.3. Assessment of Probiotic Properties of Lactic Acid Bacteria

#### 2.3.1. Acid and Bile Salt Tolerance

The lactic acid bacterial isolates were cultivated in MRS broth at 37 °C for 24 h in low-oxic conditions, after which the turbidity of the bacterial suspension was adjusted to McFarland No. 0.5 (1.5 × 10^8^ cells/mL). Then, bacterial inoculum 1% (*v*/*v*) was subjected to MRS broth pH 2.5 and MRS broth supplemented with 0.3% (*w*/*v*) bovine bile salts (Sigma-Aldrich, St. Louis, MO, USA), and then incubated at 37 °C for 6 h in low-oxic conditions using the candle jar method. Later, the suspension was serially diluted in 0.85% NaCl and spread on an MRS agar plate. After incubation at 37 °C for 24 h, colony-forming units (CFUs) of bacteria were counted and the percentage of bacterial survival was calculated. The reference probiotic strain, *Lactobacillus rhamnosus* GG (also known as LGG), was used as a positive control in the tests. The survival rate (%) was calculated using Equation (1).

(1) $$\mathrm{Survival}\;\mathrm{rate}\;(\%)\;=\frac{\mathrm{Bacterial}\;\mathrm{numbers}\;(\log\;\mathrm{CFU}/\mathrm{mL})\;\mathrm{at}\;6\;h}{\mathrm{Bacterial}\;\mathrm{number}\;(\log\;\mathrm{CFU}/\mathrm{mL})\;\mathrm{at}\;\mathrm{the}\;\mathrm{initial}\;\mathrm{time}}\;\times \;100$$

#### 2.3.2. Cell Surface Hydrophobicity

The lactic acid bacterial cells were washed three times with phosphate buffer saline (PBS) pH 7.4, and the turbidity of the cell suspension was adjusted to an OD_600_ value of 0.2 (A_0_). Then, 1 mL of suspension was mixed with an equal volume of each hydrocarbon (xylene and chloroform). The reaction mixture was mixed by vortexing and incubation at 37 °C for 1 h to separate the aqueous and hydrocarbon phases; then the aqueous phase was removed. After that, an absorbance of the hydrocarbon phase was measured at 600 nm (A_1_) and calculated for the percentage of hydrophobicity. The reference probiotic strain, *Lb. rhamnosus* GG, was used as a positive control in the tests. The hydrophobicity (%) was calculated using Equation (2).

(2) $$\mathrm{Hydrophobicity}\;(\%)\;=[1\;-\frac{A_{1}}{A_{0}}]\;\times \;100$$

#### 2.3.3. Auto-Aggregation Ability

The lactic acid bacterial cells were washed three times with PBS pH 7.4 and the suspension turbidity was adjusted to an OD_600_ value of 0.2 (A_0_). The cell suspension was mixed by vortexing and incubated at 37 °C for 4 h; then the absorbance was measured at 600 nm (A_t_) and the percentage of auto-aggregation was calculated. The reference probiotic strain, *Lb. rhamnosus* GG, was used as a positive control in the tests. The auto-aggregation (%) was calculated using Equation (3).

(3) $$\mathrm{Auto}-\mathrm{aggregation}\;(\%)\;=[1\;-\frac{A_{t}}{A_{0}}]\;\times \;100$$

#### 2.3.4. Antioxidative Activity

The scavenging of hydroxyl and 2,2-diphenyl-1-picrylhydrazyl (DPPH) free radicals was evaluated using the method modified from Sui et al. [25]. For hydroxyl radical scavenging assay, the lactic acid bacterial suspension (1.5 × 10^8^ cells/mL) was mixed with 0.75 mM 1,10-phenanthroline (Sigma-Aldrich, St. Louis, MO, USA), 0.75 mM FeSO_4_, and PBS pH 7.4. Then, 1 mL of 0.01% (*v*/*v*) hydrogen peroxide (H_2_O_2_) was added to start the reaction. After incubation at 37 °C for 1 h, an absorbance of the mixture was measured at 536 nm and the percentage of hydroxyl scavenging activity was calculated. To evaluate DPPH radical scavenging, the lactic acid bacterial suspension (1.5 × 10^8^ cells/mL) was mixed with 0.05 mM DPPH (Sigma-Aldrich, St. Louis, MO, USA). After incubation at 37 °C for 1 h in darkness, the absorbance of the mixture was measured at 517 nm and the percentage of DPPH radical scavenging activity was calculated. *Lb. rhamnosus* GG as reference probiotic strain, and butylated hydroxytoluene (BHT) as standard antioxidant agent (Sigma-Aldrich, St. Louis, MO, USA), were used as positive control in the tests. The percentage of antioxidant scavenging (%) was calculated using Equations (4) and (5).

(4) $$\mathrm{Hydroxyl}\;\mathrm{scavenging}\;(\%)\;=[\frac{\left(A_{s}\;-\;A_{c}\right)}{\left(A_{b}\;-\;A_{c}\right)}]\;\times \;100$$

where A_s_ is the absorbance of the treatment at 536 nm; A_c_ is the absorbance of the control, including 1,10-phenanthroline, FeSO_4_, and H_2_O_2_; and A_b_ is the absorbance of the blank, including 1,10-phenanthroline, and FeSO_4_.

(5) $$\mathrm{DPPH}\;\mathrm{scavenging}\;(\%)\;=[\frac{\left(A_{c}-\;A_{s}\right)}{A_{c}}]\;\times \;100$$

where A_s_ is the absorbance of the treatment at 517 nm; A_c_ is the absorbance of the control, including 2,2-diphenyl-1-picrylhydrazyl and distilled water.

### 2.4. Assay of Antimicrobial Activity Against Foodborne Pathogens

In this step, antimicrobial activity against 12 foodborne pathogens, including *E. coli* ATCC 8739, *S. aureus* ATCC 6538, *B. cereus* ATCC 11778, *S. typhimurium* ATCC 13311, *L. monocytogenes* ATCC 7644, *Proteus mirabilis* DMST 8212, *P. aeruginosa* ATCC 10145, *Shigella dysenteriae* DMST 15111, *Klebsiella pneumoniae* ATCC 13883, *Enterobacter aerogenes* ATCC 13048, *Vibrio parahaemolyticus* ATCC 17802, and *Enterococcus faecalis* ATCC 29212, was evaluated using the agar overlay technique, according to the method of Dubourg et al. [26]. The lactic acid bacterial suspension (1.5 × 10^8^ cells/mL) was dropped on MRS agar pH 6.5 and incubated at 37 °C for 24 h in low-oxic conditions using the candle jar method. After that, the 0.7% (*w*/*v*) tryptic soy broth (TSB) soft agar containing each pathogen suspension (1.5 × 10^8^ cells/mL) was overlaid on a surface of the MRS agar medium. After incubation at 37 °C for 24 h, the inhibition zone diameter (mm) was measured and the rate of pathogen inhibition was calculated. The reference probiotic strain, *Lb. rhamnosus* GG, was used as a positive control in the tests. The pathogen culture in an area of the inhibition zone was cut and placed on a coverslip. The bacterial cells were fixed in 2.5% (*v*/*v*) glutaraldehyde in 50 mM phosphate buffer pH 7.0 at 4 °C for 18 h, then washed three times with phosphate buffer pH 7.0 and dehydrated in an ethanol series. The samples were dried with CO_2_ using a Polaron Range SC7620 Critical Point Dryer (Quorum Technologies, East Sussex, UK), coated with gold particles, and then observed at 30,000× magnification in a FEI Quanta 450 scanning electron microscope (SEM; FEI Company, Hillsboro, OR, USA). The rate of pathogen inhibition was calculated using Equation (6).

(6) $$\mathrm{Rate}\;\mathrm{of}\;\mathrm{pathogen}\;\mathrm{inhibition}\;=\frac{\mathrm{Inhibition}\;\mathrm{zone}\;\mathrm{diameter}\;\mathrm{of}\;\mathrm{pathogen}\;(\mathrm{mm})}{\mathrm{Lactic}\;\mathrm{acid}\;\mathrm{bacteria}\;\mathrm{colony}\;\mathrm{diameter}\;(\mathrm{mm})}$$

### 2.5. Evaluation of Safety Profile

#### 2.5.1. Antibiotic Susceptibility

According to the method described by Chanworawit et al. [27], the antibiotic susceptibility of the isolates was evaluated using the agar disc diffusion on Mueller–Hinton agar (HiMedia Laboratories Pvt. Ltd., Mumbai, India). The lactic acid bacterial suspension (1.5 × 10^8^ cells/mL) was swabbed on the agar surface, and antibiotic discs (6 mm diameter), each containing the antibiotics ampicillin (10 µg), penicillin (6 µg), vancomycin (30 µg), kanamycin (30 µg), streptomycin (10 µg), tetracycline (30 µg), chloramphenicol (30 µg), ciprofloxacin (5 µg), and erythromycin (10 µg), were placed on the agar medium. After incubation at 37 °C for 24 h in low-oxic conditions using the candle jar method, the diameter of the inhibition zone (mm) was measured and compared with the interpretative zone diameters described in the Clinical and Laboratory Standards Institute [28].

#### 2.5.2. Hemolysin, DNase, and Biogenic Amine Production

The lactic acid bacterial culture was streaked on 5% (*v*/*v*) sheep blood agar and DNase agar (HiMedia Laboratories Pvt. Ltd., Mumbai, India). After incubation at 37 °C for 24 h in low-oxic conditions using the candle jar method, the culture plates were determined for hemolysin and DNase activity. The bacterial strain *S. aureus* ATCC 6538 was used as a positive control in the tests. To test for biogenic amine production, the bacterial inoculum (1% *v*/*v*) was subjected to modified decarboxylase medium [29], supplemented with 1% (*w*/*v*) of different amino acid precursors (histidine, tyrosine, arginine, and lysine). After incubation at 37 °C for 48 h in low-oxic conditions using the candle jar method, a change of medium color was observed.

#### 2.5.3. Detection of Virulence Factor Genes

The genomic DNA of bacterial isolate was extracted and used for detecting eight virulence factor genes (VFGs), including *ace* (collagen-binding protein), *agg* (aggregation substance), *asa*1 (aggregation substance gene), *cpd* (sex pheromone peptides), *cyl*A (cytolysin), *cyl*B (cytolysin), *efa*Afs (cell wall adhesins) and *gel*E (gelatinase), according to the PCR method of Zhang et al. [30]. Table S1 (Supplementary Materials) displays the PCR primers, reaction conditions, and product sizes. The bacterial strain *E. faecalis* ATCC 29212 was used as a positive control in the tests.

#### 2.5.4. Toxicity to Vero Cells

Cytotoxicity to an African green monkey kidney cell line (Vero) was performed using MTT (3-(4,5-dimethylthiazol-2-yl)-2,5-diphenyltetrazolium bromide) assay [31]. Vero cells were cultured in a 96-microwell plate containing Dulbecco’s Modified Eagle Medium (DMEM) (Gibco, Thermo Fisher Scientific, Waltham, MA, USA) supplemented with 10% (*v*/*v*) fetal bovine serum (FBS) (Gibco, Thermo Fisher Scientific, Waltham, MA, USA), and antibiotics (100 U/mL penicillin, and 100 µg/mL streptomycin) (Gibco, Thermo Fisher Scientific, Waltham, MA, USA) at 37 °C in an atmosphere of 5% CO_2_ and 95% O_2_. The lactic acid bacterial suspension was added into each well with different multiplicity of infection (MOI 0.1, 1, 10, and 100) and incubated at 37 °C for 24 h. Then, the well plate was washed five times with sterile 1× PBS pH 7.4, then added with 5 mg/mL MTT. The microwell plate was further incubated under the same conditions for 4 h in darkness, and then 100 µL of dimethyl sulfoxide (DMSO) was added to dissolve the MTT-formazan crystals. The solution was measured an absorbance at 570 nm using a Thermo Scientific^TM^ Multiskan GO Microplate Spectrophotometer (Thermo Fisher Scientific, Waltham, MA, USA) and the Vero cell viability (%) was calculated using Equation (7).

(7) $$\mathrm{Vero}\;\mathrm{cell}\;\mathrm{viability}\;(\%)\;=\frac{\mathrm{Absorbance}\;\mathrm{at}\;570\;\mathrm{nm}\;\mathrm{of}\;\mathrm{treatment}}{\mathrm{Absorbance}\;\mathrm{at}\;570\;\mathrm{nm}\;\mathrm{of}\;\mathrm{control}}\;\times \;100$$

### 2.6. Evaluation of Adhesion Ability of Lactic Acid Bacteria to Human Colon Adenocarcinoma Cells (Caco-2 and HT-29)

The method for evaluating adhesion ability to human colon adenocarcinoma cells was modified from Chanworawit and Deevong [32]. Human colon adenocarcinoma Caco-2 and HT-29 cells were cultured in DMEM (Gibco, Thermo Fisher Scientific, Waltham, MA, USA) supplemented with 10% (*v*/*v*) FBS (Gibco, Thermo Fisher Scientific, Waltham, MA, USA) and antibiotics (100 U/mL penicillin, and 100 µg/mL streptomycin) (Gibco, Thermo Fisher Scientific, Waltham, MA, USA) at 37 °C in an atmosphere of 5% CO_2_ and 95% O_2_. For the adherence assay, the cells were seeded in 6-well plates at the concentration of 1 × 10^6^ cells/well. The lactic acid bacterial suspension (1.5 × 10^8^ cells/mL) was added into each well and incubated at 37 °C for 1 h. The well plate was washed five times with sterile 1× PBS pH 7.4 to remove the non-adherent cells and further release the adherent cells by 1× PBS containing 0.01% (*v*/*v*) Triton X-100. The suspension was subjected to serial dilution in 0.85% (*w*/*v*) NaCl and spread on MRS agar. After incubation at 37 °C for 24 h in low-oxic conditions using the candle jar method, the bacterial colony (CFU/mL) was quantified and the percentage of adhesion was calculated. The reference probiotic strain, *Lb. rhamnosus* GG, was used as a positive control in the tests. The adherent bacteria were observed at 10,000× magnification in an FEI Quanta 450 scanning electron microscope (SEM). The adhesion ability (%) was calculated using the Equation (8).

(8) $$\mathrm{Adhesion}\;\mathrm{ability}\;(\%)\;=\frac{Adherent\;\mathrm{bacterial}\;\mathrm{numbers}\;}{\mathrm{Bacterial}\;\mathrm{number}\;\mathrm{at}\;\mathrm{the}\;\mathrm{initial}\;\mathrm{time}\;}\;\times \;100$$

### 2.7. Assay of Anti-Adhesion Ability Against Foodborne Pathogens

An anti-adhesion ability to human colon adenocarcinoma cells (Caco-2 and HT-29) based on competition assay was modified from the method of Sophatha et al. [33]. The lactic acid bacteria and foodborne pathogen suspensions (1.5 × 10^8^ cells/mL) were mixed in each well, and then incubated at 37 °C for 1 h. The well plate was washed five times with sterile 1× PBS pH 7.4 to remove the non-adherent cells and release the adherent cells by 1× PBS containing 0.01% (*v*/*v*) Triton X-100. To evaluate the anti-adhesion ability, the suspension was serially diluted in 0.85% (*w*/*v*) NaCl and spread on MacConkey agar (for *E. coli*, *P. mirabilis*, and *P. aeruginosa*), mannitol egg yolk polymyxin agar (MYPA; for *B. cereus*), mannitol salt agar (MSA; for *S. aureus*), chromogenic *Listeria* agar (CLA; for *L. monocytogenes*), *Salmonella-Shigella* agar (SSA; for *S. typhimurim* and *S. dysenteriae*), *Enterococcus* selective agar (for *E. faecalis*), and thiosulfate citrate bile salt sucrose (TCBS; for *V. parahaemolyticus*). After incubation at 37 °C for 24 h, the bacterial colony (CFU/mL) was quantified and the percentage of anti-adhesion was calculated. Competition ability (%) was calculated using the Equation (9).

(9) $$\mathrm{Competition}\;\mathrm{ability}\;(\%)\;=[1\;-\frac{\;\mathrm{pathogen}\;\mathrm{numbers}\;\mathrm{with}\;\mathrm{lactic}\;\mathrm{acid}\;\mathrm{bacteria}\;}{\mathrm{pathogen}\;\mathrm{numbers}\;\mathrm{without}\;\mathrm{lactic}\;\mathrm{acid}\;\mathrm{bacteria}}]\;\times \;100$$

### 2.8. Effect of Lactic Acid Bacteria on the Growth Performance of Two-Spotted Crickets

Healthy 14-day-old two-spotted crickets (*G. bimaculatus*) were randomly selected for the *in vivo* lactic acid bacteria feeding test. The cricket rearing method used in this study was modified from Takacs et al. [34]. The experimental units consisted of plastic boxes with a volume of 4000 cm^3^ (20 × 20 × 10 cm), containing a piece of cardboard egg tray (10 × 10 × 5 cm) and feed trays (35 × 15 mm and 60 × 15 mm Petri dish) to provide the hiding space, water, and feed sources, respectively, and each unit housed 30 crickets (*n* = 30). The rearing conditions were 70–75% relative humidity (RH) and 8:16 h light-dark (LD) cycle in a climate-controlled room at 30–35 °C. The commercial cricket feed (Pure Pride^®^ feeds; TFMs (Saraburi), Thailand) used in this study included soybean meal, corn meal, rice bran, mono-dicalcium phosphate, amino acids, vitamins, and minerals (87% dry matter, 21% crude protein, 5% fat, and 5% fiber). In the feeding experiments, the lactic acid bacteria were added to the cricket feed at the final concentration of 10^9^ cells/g feed, and the feed was replaced every 48 h. An experiment that excluded the addition of lactic acid bacteria to the feed functioned as a control in the tests. To avoid microbial contamination during insect sampling, the experimental groups for the different feeding durations of 7 and 14 days were segregated, with each group including five replicates (5 boxes containing 30 insects each). Dead crickets found in the containers were eliminated to prevent cannibalism. At 1 and 2 weeks of feeding, the survival rate (%), body weight (g), and body length (mm) of the crickets from the test and control groups were determined. Moreover, a total of 50 crickets were randomly sampled from all experimental units (10 crickets per box) and dried at 60 °C for 24 h; then the milled sample was used for evaluating chemical composition using proximate analysis according to the method of Chanadang et al. [35], including crude protein (CP), ash, crude fiber (CF), and crude fat.

### 2.9. Analysis of Bacterial Community by 16S Metagenomics Long-Read Sequencing

At 7 and 14 days of the feeding experiments, 30 crickets were randomly taken from every experimental unit (6 crickets per box). The insect surface was washed three times with sterile distilled water. On an ice-cold plate, insect gut was dissected and the gut content was homogenized in phosphate buffered saline (PBS) pH 7.4 with a sterile pestle and used for DNA extraction. Genomic DNA of the cricket guts was extracted using the ZymoBIOMICS^®^ DNA Kit (Zymo Research, Irvine, CA, USA) according to the manufacturer’s instruction and quantified using a NanoDrop 2000 spectrophotometer (Thermo Fisher Scientific, Waltham, MA, USA). The V1–V9 regions of the 16S rRNA gene was amplified by the primer pair with specific PacBio barcode sequences: 27F (AGRGTTTGATYNTGGCTCAG) and 1492R (TASGGHTACCTTGTTASGACTT). The PCR reaction was performed by the KAPA HiFi Hot Start DNA polymerase (KAPA Biosystems, Wilmington, MA, USA). The amplified products from each sample were pooled, and the DNA library was constructed using the SMRTbell Express Template Prep Kit (Pacific Biosciences, Menlo Park, CA, USA). The library was quantified using a Qubit Fluorometer (Invitrogen, Thermo Fisher Scientific, Waltham, MA, USA) and sequencing on the PacBio Sequel II system (Pacific Biosciences, Menlo Park, CA, USA). The demultiplexed sequence data in FASTQ format was primarily processed using Trimmomatic version 0.33, removal of the primer sequence using Cutadapt version 1.9.1, assembly of the paired-end reads using USEARCH version 10.0, and chimera removal using UCHIME version 8.1. The operational taxonomic units (OTUs) were applied to cluster reads with similarity above 97.0%, using USEARCH software (version 10.0). The taxonomic annotation of feature sequences was processed by nucleotide BLAST using the 16S full-length database of Silva version 138.1 and Greengenes version 13.5. Downstream visualization was performed in R version 4.5.2 (R Foundation for Statistical Computing, Vienna, Austria). Alpha (α)-diversity indices (Shannon index) were calculated and rarefaction curves were generated using the phyloseq and vegan packages, Beta (β)-diversity was assessed based on Bray–Curtis dissimilarity and visualized using Principal Coordinates Analysis (PCoA) and non-metric multidimensional scaling (NMDS) implemented in the vegan package, and Venn diagrams illustrating shared and unique OTUs among groups were constructed using the Venn Diagram package. All graphical outputs were generated using ggplot2.

### 2.10. Statistical Analysis and Accession Number

All tests were performed at least in triplicate. The results are expressed as mean ± standard deviation (S.D.). The data were analyzed using one-way ANOVA, followed by Duncan’s multiple range post hoc test, using SPSS version 25.0 (IBM Corp., Armonk, NY, USA). Normality and homogeneity of variance were assessed using the Shapiro-Wilk test and Levene’s test, respectively. Differences were considered statistically significant at *p* < 0.05. The nucleotide sequences of the selected lactic acid bacteria (TPL-1 to TPL-5) and the negative reference strain TPL-S have been available at the GenBank database under the accession numbers of PX963459–PX963464, respectively.

## 3. Results

### 3.1. Isolation and Identification of Lactic Acid Bacteria from the Guts of Termites

**Figure 1:**
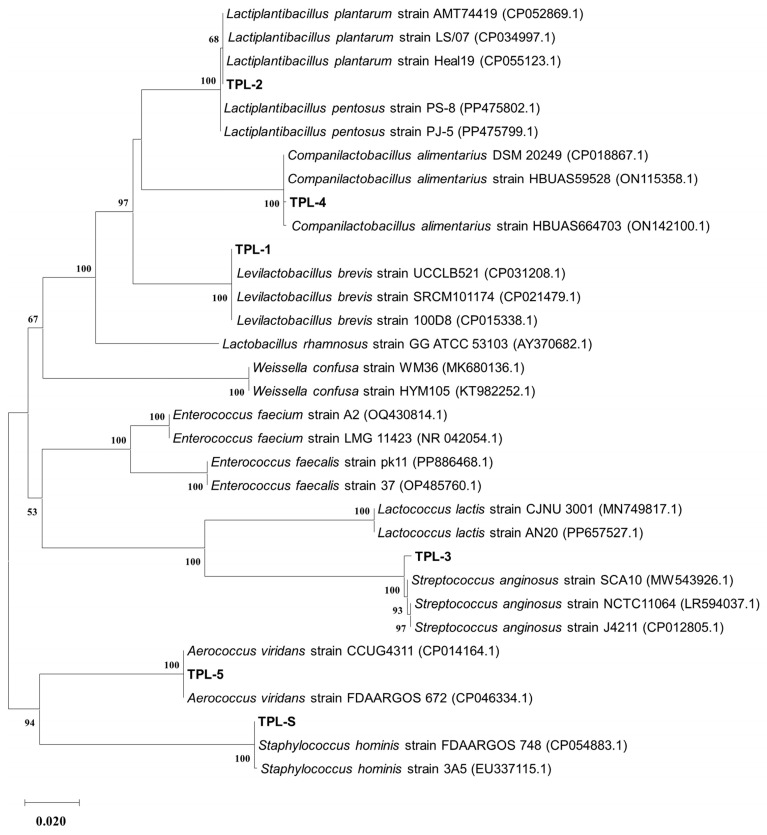
Phylogenetic tree of 16S rRNA gene sequences of the selected lactic acid bacteria (TPL-1 to TPL-5) and negative reference strain TPL-S isolated from guts of the termite *Termes propinquus*. The tree was constructed by MEGA11 software after multiple sequence alignments of the data by ClustalW. Numbers at the nodes indicate the level of bootstrap (%) based on a maximum-likelihood method analysis of 1000 resampled datasets, and the evolutionary distances were computed using the Kimura two-parameter method. The scale bar represented 0.02 nucleotide substitution per sequence position.

In the bacterial isolation step, a total of 49 isolates of morphologically different bacteria were isolated from the guts of the termite *T. propinquus*. In the total number, five isolates (designated as TPL-1 to TPL-5) of catalase-negative, Gram-positive non-endospore forming rods and cocci were selected for the subsequent experiments. Based on nucleotide sequencing of 16S rRNA genes, the isolates TPL-1 to TPL-5 were closely related to *Lb. brevis* UCCLB521 (CP031208.1) with 100% identity, *Lb. plantarum* Heal19 (CP055123.1) with 100% identity, *S. anginosus* SCA10 (MW543926.1) with 99.67% identity, *C. alimentarius* DSM 20249 (CP018867.1) with 99.93% identity, and *A. viridans* CCUG4311 (CP014164.1) with 100% identity, respectively. Among the excluded 44 isolates of Gram-positive catalase-positive cocci, only one isolate (designated as TPL-S) was randomly picked and used as a negative reference strain. The molecular identification demonstrated that the nucleotide sequence of TPL-S was 100% identical to the pathogenic bacteria *S. hominis* FDAARGOS (CP054883.1). The phylogenetic tree based on 16S rRNA nucleotide gene sequences of the isolates clustered with lactic acid bacteria sequences from the NCBI GenBank database is shown in Figure 1. The bacterial identification using VITEK^®^MS (MALDI-TOF) revealed that the isolates TPL-1 to TPL-5 were identified as *Lb. brevis*, *Lb. plantarum*, *S. anginosus*, *Lb. alimentarius*, and *A. viridans*, respectively.

### 3.2. Probiotic Properties of Lactic Acid Bacteria

**Figure 2:**
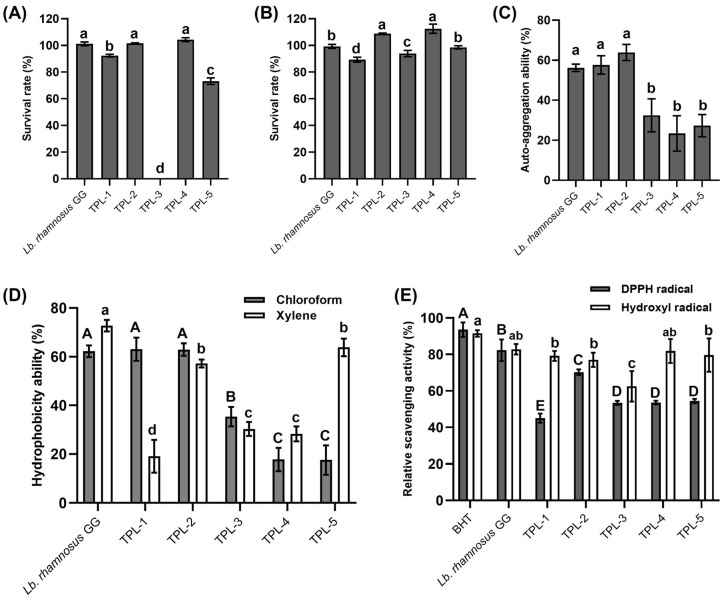
Probiotic properties of the selected lactic acid bacteria (TPL-1 to TPL-5) based on survival in pH 2.5 (**A**), and 0.3% bile salts (**B**); auto-aggregation (**C**); cell surface hydrophobicity (**D**); and antioxidant activity (**E**). Each value represents the mean ± S.D. of triplicate determinations. Statistical significances were determined using one-way ANOVA in SPSS v. 25.0, and the different superscript letters indicate significant difference (*p* < 0.05) within the experimental group. *Lactobacillus rhamnosus* GG as reference probiotic strain and butylated hydroxytoluene (BHT) as standard antioxidant agent were used in the tests.

The five chosen isolates were accessed for tolerance to acid and bile salts, bacterial cell-surface characteristics, and antioxidant activity. The probiotic properties of the isolates and reference probiotic strain are shown in Figure 2. In the investigation of acid resistance, *S. anginosus* TPL-3 could not survive at pH 2.5 after 6 h of incubation; the remaining isolates exhibited good survival, with 73.16–104.31%. In addition to their acidity tolerance, *Lb. plantarum* TPL-2 (101.57 ± 0.34% survival) and *C. alimentarius* TPL-4 (104.31 ± 1.48% survival) exhibited an ability to grow in the acidic conditions, exhibiting a slightly higher value in comparison to the reference probiotic strain *Lb. rhamnosus* GG (101.15 ± 1.43% survival). All five isolates showed 89.31–112.48% survival at the concentration of 0.3% bile salts after 6 h of incubation. Interestingly, *Lb. plantarum* TPL-2 (108.71 ± 0.52% survival) and *C. alimentarius* TPL-4 (112.48 ± 3.45% survival) showed the significantly highest bile salt tolerance, which was higher than that of *Lb. rhamnosus* GG (99.27 ± 1.53% survival) (Figure 2A,B).

The cell-surface characteristics of lactic acid bacteria based on auto-aggregation and hydrophobicity were investigated, and the results are shown in Figure 2C,D. The isolates showed an auto-aggregation ability ranging from 23.41–63.85% after 4 h of incubation, and *Lb. plantarum* TPL-2 exhibited the highest auto-aggregation ability value, with 63.85 ± 4.02%. Moreover, all isolates presented cell-surface hydrophobicity in all two solvents. *Lb. brevis* TPL-1 (63.07 ± 4.79%) and *Lb. plantarum* TPL-2 (62.90 ± 2.57%) showed the significantly highest hydrophobicity values over 62% in chloroform, comparable to *Lb. rhamnosus* GG (62.26 ± 2.40%). Among all isolates, *Lb. plantarum* TPL-2 and *A. viridans* TPL-5 showed the significantly highest hydrophobicity values of 57.19 ± 1.65% and 63.80 ± 3.66% in xylene, respectively, which were inferior to those of the reference probiotic strain *Lb. rhamnosus* GG (72.73 ± 2.37%).

The hydroxyl radical and DPPH radical scavenging ability of the isolates were compared with *Lb. rhamnosus* GG as reference probiotic strain and butylated hydroxytoluene (BHT) as standard antioxidant agent (Figure 2E). All isolates had hydroxyl radical and DPPH radical scavenging ability in the relative scavenging activity range of 62.50–81.90% and 45.06–70.21%, respectively. However, BHT showed the highest values of hydroxyl and DPPH radical scavenging activity with 91.51 ± 1.79% and 93.60 ± 3.94%, respectively. *Lb. rhamnosus* GG (82.29 ± 5.92%) exhibited significantly higher DPPH radical scavenging value than all isolates, while its hydroxyl scavenging ability was comparable to that of *C. alimentarius* TPL-4, with approximately 82% relative scavenging activity.

### 3.3. Antimicrobial Activity of Lactic Acid Bacteria Against Foodborne Pathogens

**Figure 3:**
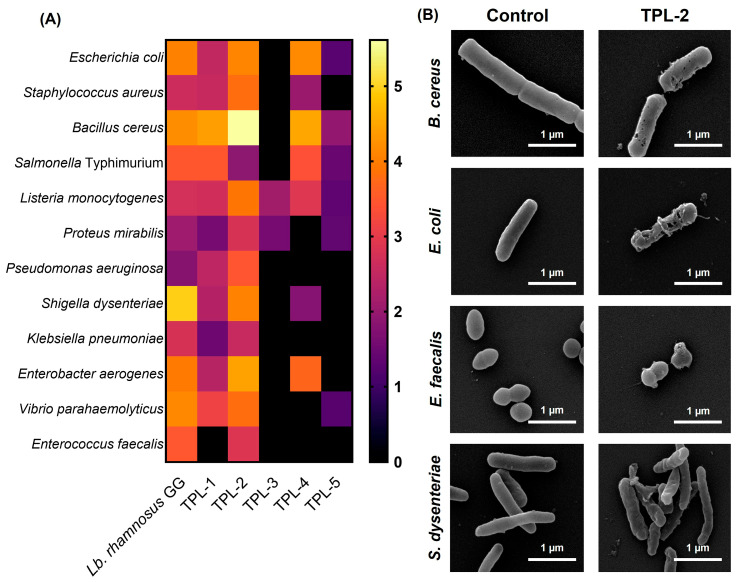
Heat map analysis of antimicrobial activity against 12 foodborne pathogens of lactic acid bacteria (TPL-1 to TPL-5), with color intensity indicating rate of inhibition (**A**) and SEM micrographs showing cell morphology of the foodborne pathogens in the *Lactiplantibacillus plantarum* TPL-2 treatment and controlled experiments (**B**). A scale bar indicates a 1-µm length.

The antimicrobial activity against 12 foodborne pathogens was evaluated using the agar overlay technique. As shown in Figure 3, all isolates inhibited *L. monocytogenes* ATCC 7644 in the inhibition range of 1.31–3.89. Among them, *Lb. plantarum* TPL-2 showed broad-spectrum antibacterial activity against all tested pathogens. TPL-2 exhibited the highest inhibition values against *E. coli* ATCC 8739, *S. aureus* ATCC 6538, *B. cereus* ATCC 11778, *L. monocytogenes* ATCC 7644, *P. mirabilis* DMST 8212, *P. aeruginosa* ATCC 10145, and *E. aerogenes* ATCC 29212, whereas *S. anginosus* TPL-3 demonstrated lowest antibacterial effectiveness. After the inhibition, cell morphological changes of foodborne pathogens were visualized under a scanning electron microscope. Compared to the control (without treatment), the foodborne pathogens had cell membrane damage, disrupted cell walls, and irregular cell morphology. The percentage of damaged cells was determined by evaluating 10 independent microscopic fields, and the reported values represent the mean proportion of affected cells. The image shown in Figure 3B is a representative field in which >80% of the cells exhibited morphological damage, consistent with the overall observations.

### 3.4. Safety Profile of Lactic Acid Bacteria

**Figure 4:**
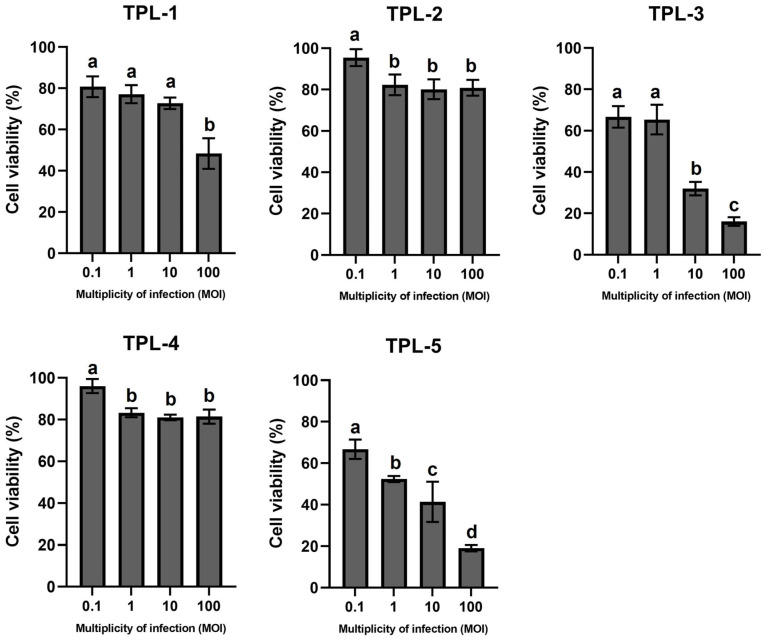
Cell viability (%) of normal kidney of African green monkey (Vero) cells after adding with lactic acid bacteria (TPL-1 to TPL-5) at different multiplicity of infection (MOI 0.1, 1, 10, and 100) and incubated at 37 °C for 24 h. Each value represents the mean ± S.D. of triplicate determinations. Statistical significances were determined using one-way ANOVA in SPSS v. 25.0, and the different superscript letters indicate significant difference (*p* < 0.05) within the experimental group.

The safety evaluations of lactic acid bacteria encompassed assessments of antibiotic susceptibility, hemolysin production, DNase activity, biogenic amine synthesis, identification of virulence factor genes, and cytotoxicity. As shown in Table 1, all selected isolates were resistant or intermediate to three antibiotics (penicillin, kanamycin, and streptomycin) and showed different antibiotic susceptibility patterns to other six antibiotics (ampicillin, vancomycin, tetracycline, chloramphenicol, ciprofloxacin, and erythromycin). Chloramphenicol was the most effective antibiotic, whereas penicillin had a low effect on all isolates. All isolates exhibited alpha-hemolysis, except for *Lb. brevis* TPL-1 and *Lb. plantarum* TPL-2, which exhibited gamma-hemolysis; none of the isolates produced the DNase enzyme. Moreover, the screening of the major biogenic amines (histamine, tyramine, putrescine, and cadaverine) revealed that all isolates did not produce detectable amines, with the exception of *Lb. brevis* TPL-1, which produced histamine, and putrescine and *A. viridans* TPL-5, which produced histamine, and cadaverine. The detection of major putative virulence factor genes (VFGs) of lactic acid bacteria was performed by polymerase chain reaction. Comparable to the positive control (*E. faecalis* ATCC 29212), all examined virulence factor genes were not presented in *Lb. plantarum* TPL-2 and *C. alimentarius* TPL-4. However, *Lb. brevis* TPL-1 harbored *cpd* (sex pheromone peptides), and *cyl*A (cytolysin) genes; *S. anginosus* TPL-3 had *ace* (collagen-binding protein) and *gel*E (gelatinase) genes; and *A. viridans* TPL-5 had only the *asa*1 (aggregation substance) gene. The toxicity of lactic acid bacteria to normal kidney of African green monkey (Vero) cells is shown in Figure 4. Among all isolates, *Lb. plantarum* TPL-2 and *C. alimentarius* TPL-4 showed non- or weak toxicity to Vero cells (>75% Vero cell viability) at all dose levels (MOI 0.1–100), while *Lb. brevis* TPL-1 showed moderate toxicity (>60% Vero cell viability at MOI 0.1–10 and <60% Vero cell viability at MOI 100). However, *S. anginosus* TPL-3 and *A. viridans* TPL-5 showed strong toxicity at high doses of bacterial concentrations (MOI 10 and 100), with <40% Vero cell viability.

### 3.5. Adhesion Ability of Lactic Acid Bacteria to Human Colon Adenocarcinoma Cells

**Figure 5:**
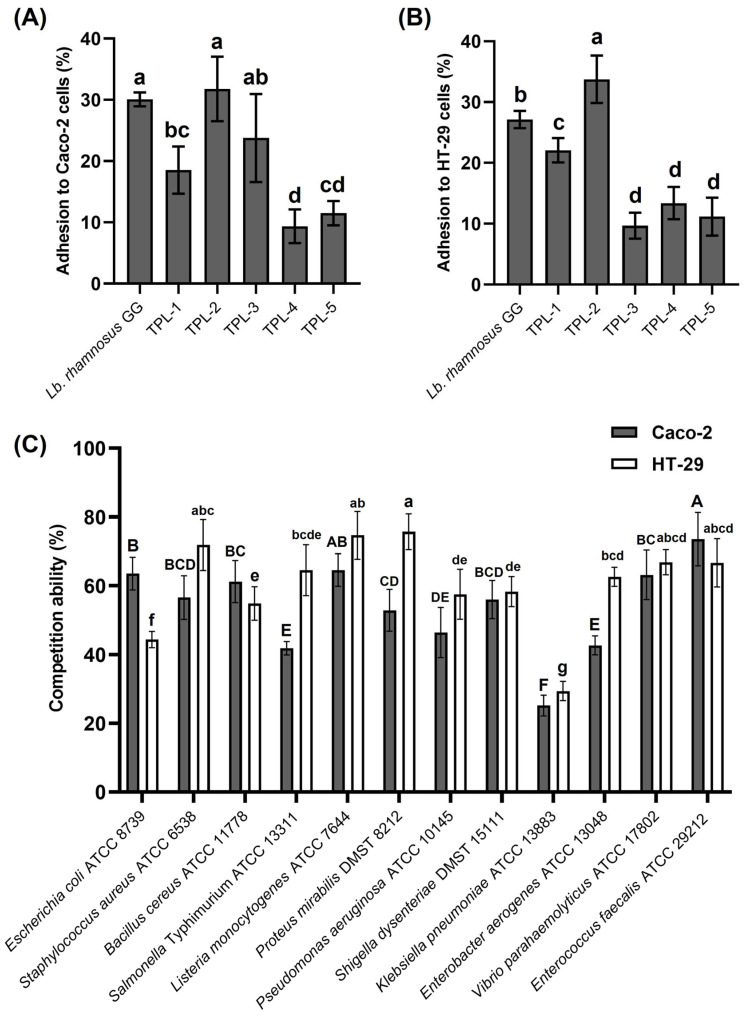
Adhesion ability (%) of lactic acid bacteria (TPL-1 to TPL-5) to human colon adenocarcinoma cells Caco-2 (**A**) and HT-29 (**B**), and competition ability (%) of *Lactiplantibacillus plantarum* TPL-2 against 12 foodborne pathogens (**C**). The probiotic strain *Lactobacillus rhamnosus* GG was used as positive control in the tests. Each value represents the mean ± S.D. of triplicate determinations. Statistical significances were determined using one-way ANOVA in SPSS v. 25.0, and the different superscript letters indicate significant difference (*p* < 0.05) within the experimental group.

**Figure 6:**
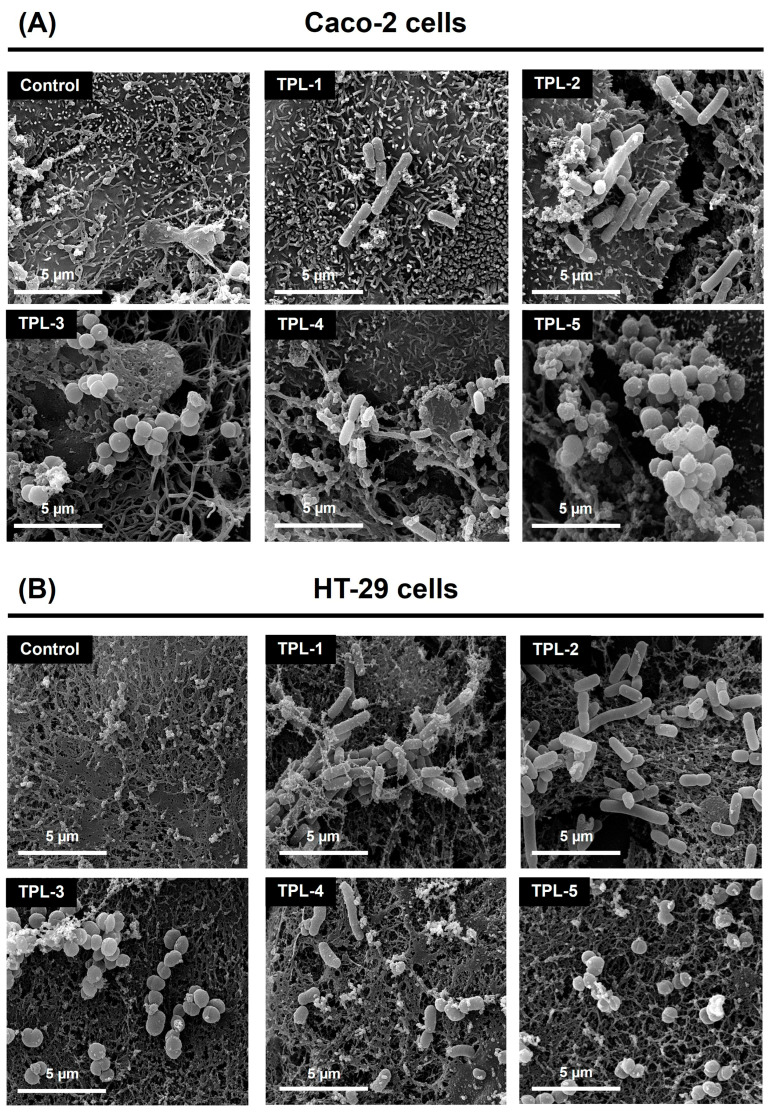
SEM microphotographs showing adherent cells of lactic acid bacteria (TPL-1 to TPL-5) to human colon adenocarcinoma cells Caco-2 (**A**) and HT-29 (**B**) at 10,000× magnification. A scale bar indicates a 5-µm length.

The adhesion capability to two human colon adenocarcinoma cells, Caco-2 and HT-29, of lactic acid bacteria was evaluated in this step. The adhesion index (%) among isolates ranged from 9.36% to 31.80% to Caco-2 cells, and 9.68% to 33.75% to HT-29 cells. Among all lactic acid bacteria, *Lb. plantarum* TPL-2 had the highest adhesion capacity to both Caco-2 and HT-29 cells, with adhesion percentages of 31.80 ± 5.27% and 33.75 ± 3.89%, respectively, surpassing that of the reference probiotic strain *Lb. rhamnosus* GG (Figure 5A,B). In contrast, *C. alimentarius* TPL-4 exhibited the lowest adhesion ability to Caco-2 (9.36 ± 2.75% adhesion), and *S. anginosus* TPL-3 showed the lowest adhesion ability to HT-29 (9.68 ± 2.15% adhesion). The visualizations showing adherent cells of lactic acid bacteria to the human colon adenocarcinoma cells, Caco-2 and HT-29, under SEM at 10,000× magnification, are shown in Figure 6. The promising *Lb. plantarum* TPL-2 was selected for further experiments.

### 3.6. Anti-Adhesion Ability of Lactic Acid Bacteria Against Foodborne Pathogens

The evaluated anti-adhesion ability effects on two human colon adenocarcinoma cells, Caco-2 and HT-29, of *Lb. plantarum* TPL-2 against 12 foodborne pathogens using the competitive ability assay are shown in Figure 5C. The competition ability assay was carried out, and *Lb. plantarum* TPL-2 displayed the most competitive capacity against all tested pathogens, with over 40%, with the exception of *K. pneumoniae* ATCC 13883. The potential strain TPL-2 exhibited the highest competitive ability against *E. faecalis* ATCC 29212, with 73.55 ± 7.74% to Caco-2 cells, and *P. mirabilis* DMST 8212, with 75.71 ± 5.20% to HT-29 cells, whereas it showed the minimum competitive adhesion against *K. pneumoniae* ATCC 13883, with 25.15 ± 3.04% to Caco-2 cells and 29.37 ± 2.79% to HT-29 cells.

### 3.7. Effect of Lactic Acid Bacteria Supplementation on the Growth Parameters and Gut Microbiota Homeostasis of Two-Spotted Crickets

**Figure 7:**
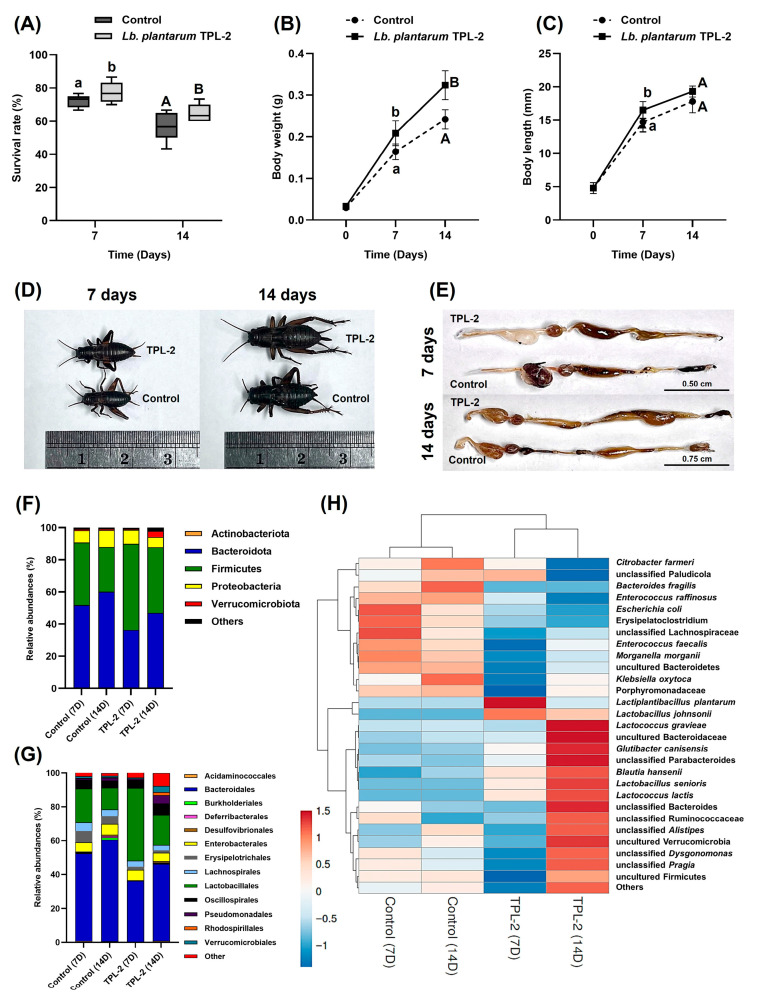
Effects of *Lactiplantibacillus plantarum* TPL-2 on the two-spotted crickets (*Gryllus bimaculatus*) and intestinal bacterial microbiome in control and treatments at 7 and 14 days of feeding: insect survival (%) (**A**), body weight (g) (**B**), and body length (mm) (**C**); photographs of insect bodies (**D**), and whole guts (**E**); the relative abundance (%) of bacterial phylum (**F**) and order (**G**) in the cricket guts, based on the 16S rRNA gene-based metagenomic analysis and heat map showing relative abundance in species level of bacteria, with color intensity indicating abundance (**H**). Each value in (**A**–**C**) represents the mean ± S.D. of triplicate determinations. Statistical significances were determined using one-way ANOVA in SPSS v. 25.0, and the different superscript letters indicate significant difference (*p* < 0.05) within the experimental group.

In this step, the promising strain *Lb. plantarum* TPL-2 was added to the feed for 14-day-old crickets. During the treatment period, the feed was replaced every 48 h and the cricket samples were collected at 7 and 14 days for experiments. The results demonstrated the effects of *Lb. plantarum* TPL-2 on the two-spotted crickets (*G. bimaculatus*) and their gut bacterial microbiome. The insect survival (%), body weight (g), and length (mm) of crickets are shown in Figure 7. The survival percentages of the crickets treated with *Lb. plantarum* TPL-2 were 77.33 ± 6.41% and 64.67 ± 5.58% at 7 and 14 days of treatment, respectively, surpassing that of the control (72.00 ± 3.80% and 57.33 ± 8.95% survival, respectively. The crickets fed with *Lb. plantarum* TPL-2 showed a higher body weight and body length compared to the control, and their gut compartments are represented in Figure 7E. Additionally, the proximate analysis of nutritional composition of crickets, specifically the major components including protein, fat, ash, and fiber, is shown in Table 2. Almost all nutrient compounds were increased, except for crude fat and fiber, after 7 days of treatment. The content of crude fat was significantly reduced at 7 days of treatment, with approximately 17.39% decrease. Compared to the control group, the content of ash and crude protein in *Lb. plantarum* TPL-2 treatment group was significantly increased at 7 and 14 days of treatment, and the highest increased value (9.61% increase) of crude protein content was found at 7 days of treatment.

The effects of *Lb. plantarum* TPL-2 on the gut microbial communities in crickets were estimated using full-length 16S rRNA gene sequences obtained through PacBio long-read DNA sequencing and are shown in Figure 7F–H. After feeding with *Lb. plantarum* TPL-2, the overall bacterial community in the cricket gut from treatment group was changed. Compared to the control group, the relative abundance of phylum Firmicutes increased, while the population of Bacteroidetes (in Bacteroidota) decreased. In addition, some phyla, including Actinobacteriota and Verrucomicrobiota, decreased at 7 days and slightly increased at 14 days, while Proteobacteria increased at 7 days and decreased at 14 days. At the species level (Figure 7H), the relative abundance of *Lb. plantarum* significantly increased in the treatment group, and its maximum abundance was found at 7 days of treatment. Moreover, *Lb. plantarum* TPL-2 in the treatment group significantly promoted the number of beneficial probiotic bacteria in the cricket guts, such as *Lb. johnsonii*, *Lb. senioris* and *L. lactis*. Compared to the control group, the treatment group showed a decrease in the relative abundance of various pathogenic bacteria, such as *Citrobacter farmeri*, *E. faecalis*, *E. raffinosus, E. coli*, *K. oxytoca*, and *Morganella morganii*. These results indicated that the promising strain *Lb. plantarum* TPL-2 had an ability to improve insect viability, growth, and nutrient composition and reduce pathogen populations in the cricket samples.

**Figure 8:**
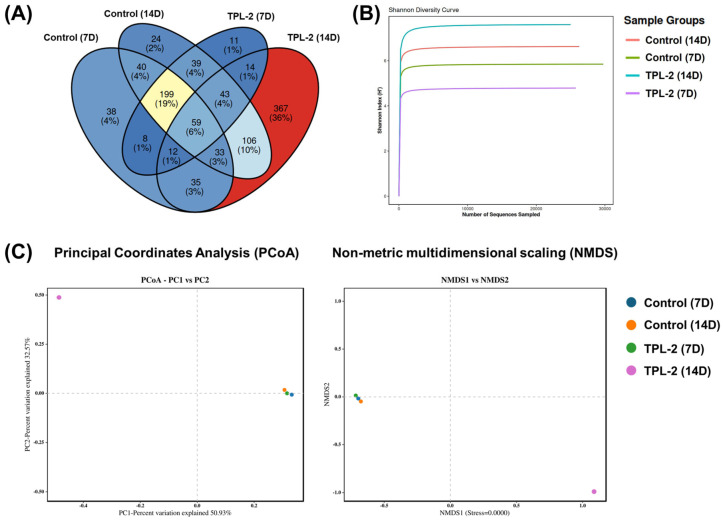
Microbial diversity and OTU distribution among experimental groups. (**A**) Venn diagram showing shared and unique OTUs among control and TPL-2 treatment, (**B**) Shannon diversity rarefaction curves showing α-diversity across groups of microbial communities, and (**C**) β-diversity analysis of microbial communities based on Principal Coordinates Analysis (PCoA) and Non-metric multidimensional scaling (NMDS) plots.

A Venn diagram was constructed to compare shared and unique OTUs among the four experimental groups of control and TPL-2 treatment. A total of 199 OTUs (19%) were shared across all groups, representing the core microbiota. The highest number of unique OTUs was observed in the TPL-2 (14 days) group (367 OTUs, 36%), indicating a pronounced shift in microbial composition following prolonged TPL-2 treatment. Moderate overlap was observed between time-matched groups and treatment-matched groups, suggesting that both treatment and duration influenced microbial community structure (Figure 8A). Rarefaction analysis demonstrated that all curves reached a plateau, indicating sufficient sequencing depth to capture microbial community diversity. Among the groups, TPL-2 at 14 days exhibited the highest Shannon diversity index, followed by control at 14 days, control at 7 days, and TPL-2 at 7 days, respectively. These findings suggest that prolonged TPL-2 treatment may enhance microbial diversity, whereas short-term exposure appears to reduce community evenness and richness. Consistent with these results, the species-level heat map revealed that treatment with TPL-2 at 14 days markedly increased the relative abundance of lactic acid bacteria. Additionally, β-diversity analysis based on Bray–Curtis dissimilarities revealed clear differences in microbial community composition among groups. The PCoA plot showed that samples were distinctly separated along PC1 (50.93%) and PC2 (32.57%), with the TPL-2 (14 days) group clearly segregating from the other groups. Similarly, NMDS analysis demonstrated distinct clustering patterns, supporting substantial community shifts following prolonged TPL-2 treatment (Figure 8B,C).

## 4. Discussion

Our present study provided knowledge of the probiotic properties of lactic acid bacteria derived from the guts of the termite *T. propinquus* and application to enhance the growth and gut microbiome of the widely consumed cricket *G. bimaculatus*. Similarly, lactic acid bacteria are commonly found in various insect species, including honeybees, ants, silkworms, termites, and bean bugs [5,36,37,38,39]. In this present study, a total of five lactic acid bacteria (TPL-1 to TPL-5) were selected and identified as *Lb. brevis*, *Lb. plantarum*, *S. anginosus*, *C. alimentarius*, and *A. viridans*, respectively. These bacterial species have been isolated from various sources, for example *C. alimentarius* as a facultatively heterofermentative lactic acid bacterium from spontaneous fermented sausages [40], potential probiotic *Lb. brevis* L010 from Korean fermented Jangajji [41], *Lb. plantarum* MKTJ24 from an artisanal fermented fish of Northeast India [42], and probiotic *A. viridans* LL1 from indoor dust collected from a patient’s home [43]. The pathogenic *S. milleri* group, including *S. anginosus*, *S. constellatus,* and *S. intermedius*, were commonly isolated from the human body, primarily in the oral cavity, upper respiratory tract, gastrointestinal tract, and urogenital tract [44].

*In vitro* determinations are commonly used for probiotic evaluations, which include inhibiting the growth of harmful microorganisms, modulating the immune system, survival in the digestive and gastrointestinal tracts, adhesion to intestinal surfaces, safety, potentially reducing the risk of certain diseases, and producing beneficial compounds. In the present study, all five isolates, except *S. anginosus* TPL-3, could tolerate the harsh conditions of the digestive system with a high acidity at pH 2.5 (73.16–104.31% survival) and the value of 0.3% bile salts (89.31–112.48% survival). The tolerance ability to the stressful conditions of lactic acid bacteria is crucial for their survival and potential probiotic benefits. The resistant strains can transit through the stomach and small intestine to reach the colon in a viable state, where they can potentially exert their beneficial effects [45]. Similarly, the reported *Lactobacillus* species, such as *Lb. plantarum*, *Lb. casei*, *Lb. rhamnosus*, and *Lb. acidophilus*, could survive in gastrointestinal tract with stressful conditions of low pH and bile salts [46,47]. Moreover, the isolates in the present study displayed antioxidant activities to DPPH and H_2_O_2_, which can help to protect the host from oxidative damage associated with various diseases. Bryukhanov et al. [48] reported that *Lactobacillus* spp. showed high antioxidant potential by production of antioxidant enzymes, such as NADH oxidase, NADH peroxidase, catalase, superoxide dismutase, and thioredoxin reductase. In addition, diverse lactic acid bacterial strains can exhibit antimicrobial activity against pathogenic microorganisms, particularly foodborne pathogens, primarily through the production of various antimicrobial compounds. These bioactive compounds can inhibit or kill pathogens, making the lactic acid bacteria useful as potential agents in biological control applications. In the present study, all strains, especially *Lb. plantarum* TPL-2, showed broad-spectrum antibacterial activities against all tested foodborne pathogenic bacteria, comparable to the probiotic reference strain *Lb. rhamnosus* GG. Members of *Lactobacillus* species have been reported as biological control agents against diverse pathogens and alternative tools to antibiotics in humans and animals. In previous reports, *Lb. plantarum* strains P1, S11, and M7 had strong antimicrobial activity against pathogenic bacteria (*S. aureus* ATCC 12600, *E. coli* ATCC 35128, and *Salmonella* sp. ASI 1174) [49], and *Lactobacillus* spp. isolated from fermented foods exhibited inhibitory effect against foodborne pathogens, including *S. aureus*, *E. coli*, and *Salmonella* spp. [50]. In addition, *Streptococcus* spp., such as *S. thermophilus* strains KKUPA22 and KKUPK13, exhibited antimicrobial activity against pathogenic bacteria [51].

Safety assessments of lactic acid bacteria, concerning the antibiotic susceptibility test, harmful substance production, identification of potential virulence factor genes, and toxicity to normal cells, are crucial to ensure the safe use of strains, especially in vulnerable populations, by identifying and mitigating potential risks associated with their probiotic or starter culture applications [52]. In the present study, all strains were susceptible to chloramphenicol (an amphenicol antibiotic). Antibiotic susceptibility testing to widely used antibiotics is crucial for identifying antibiotic-resistant strains, selecting effective antibiotic agents against bacterial infections, and evaluating the efficacy of new antimicrobials. In terms of harmful ability, two strains (*Lb. brevis* TPL-1 and *Lb. plantarum* TPL-2) showed gamma hemolysis, and the other three strains exhibited weak alpha hemolysis. All five strains were unable to produce DNase, and three strains, including *Lb. plantarum* TPL-2, *S. anginosus* TPL-3, and *C. alimentarius* TPL-4, were incapable of synthesizing major biogenic amines (histamine, tyramine, putrescine, and cadaverine). However, *Lb. brevis* TPL-1 could produce histamine and putrescine, and both histamine and cadaverine were synthesized by *A. viridans* TPL-5. Similarly, Russo et al. [53] reported that *Lb. brevis* IOEB 9809 could produce biogenic amines (BAs), including tyramine and putrescine, through amino acid decarboxylation, which can be a concern in fermented foods due to potential toxicity. In addition, *Lb. plantarum* TPL-2 and *C. alimentarius* TPL-4 revealed negative results for all virulence factor genes (VFGs), indicating their safety. In the study reported by Zhang et al. [30], lactic acid bacteria were isolated from the vagina of yak (*Bos grunniens*), and the virulence factor genes were determined. In the report, isolates *Leuconostoc mesenteroides* YD6 and *Enterococcus hirae* YD14 did not present the virulence genes, including *ace* (collagen-binding protein), *agg* (aggregation substance), *asa*1 (aggregation substance gene), *cpd* (sex pheromone peptides), *cyl*A (cytolysin), *cyl*B (cytolysin), *efa*Afs (cell wall adhesins), and *gel*E (gelatinase). As a widely used method, the cytotoxicity assay has been used to evaluate the harmful effects of substances on living cells. The normal kidney Vero cells are commonly used to evaluate the toxicity of various substances produced by microorganisms, including lactic acid bacteria. In the present study, the different cell concentrations (MOI 0.1, 1, 10, and 100) of *Lb. plantarum* TPL-2 and *C. alimentarius* TPL-4 displayed non-cytotoxicity, with the highest percentage of Vero cell viability (>80%). According to ISO 10993-5:2009, to assess the *in vitro* cytotoxicity, cell viability percentages of more than 80% indicate as non-cytotoxicity; 80–60% as low cytotoxicity; 60–40% as moderate cytotoxicity; and below 40% as high cytotoxicity [54]. Similarly to the previous study, the lactic acid bacteria *E. faecalis* MSMC104-2 and MSMC111-2 have been shown to be nontoxic to Vero cells at the cell viability percentages of 95.72 ± 3.97% and 98.85 ± 3.90%, respectively [31].

The adhesion ability of lactic acid bacteria to human colon epithelial cells is a crucial characteristic for probiotic bacteria, impacting their ability to exert beneficial effects. An adherence of probiotic bacteria to the intestinal cells can prevent pathogen adhesion, modulate the immune system, and promote overall gut health. Several factors influence this adhesion process, including bacterial surface molecules, host cell receptors, and environmental conditions. In the present study, all strains showed the adhesion ability to human colon adenocarcinoma cells (Caco-2 and HT-29) in a range of 9.36% to 33.75% adhesion. Comparable results have been reported for *S. thermophilus* JAMI_LB_02, which showed adhesion ability to Caco-2 cells at 2.10 ± 0.94% and to HT-29 cells at 3.32 ± 0.38%. However, *Lb. plantarum* JAMI_LB_05 achieved the higher adhesion percentage of 5.62 ± 1.33% to Caco-2 and 5.76 ± 0.46% to HT-29 cells [55]. Probiotic bacteria possess anti-adhesion properties that are beneficial in preventing the attachment of harmful bacteria to the host cell surface. In the present study, the high-potential strain *Lb. plantarum* TPL-2 was selected for the study of anti-adhesion ability based on the competition assay. This strain presented strong anti-adhesion ability to Caco-2 and HT-29 cells against all tested pathogens, especially *E. faecalis* and *P. mirabilis*, respectively. In the previous report, *Lb. plantarum* strain 0743 was able to reduce *Salmonella* adhesion to Caco-2 and HT-29 cells, and *Lb. paracasei* CCMA 0505 presented 4.75% adhesion to Caco-2 cells and showed a competitive effect against *E. coli* adhesion to HT-29 cells [56].

Insects are known to associate with beneficial microorganisms that contribute to host nutrition, immunity, and protection against pathogens and environmental stress. Many insect gut microbes are acquired from environmental sources such as soil, diet, or habitat, and can enhance host fitness and survival [57,58,59]. Therefore, the beneficial effects of termite-derived LAB observed in this study are consistent with the ecological role of environmentally acquired probiotics in improving insect gut microbiota and their growth performance. For the gut microbiota analysis in the current study, comparing different experimental groups within a single cricket species sourced from a singular location may be inadequate because the gut microbial community is heavily influenced by animal genetics, nutrition provided, rearing management, and environmental conditions. Among these factors, supplementation of the probiotic *Lb. plantarum* TPL-2 into feed may be the primary factor affecting the gut bacterial ecology and production performance of the two-spotted cricket (*G. bimaculatus*). The study serves as crucial background information for further applications.

In our study, *Lb. plantarum* TPL-2 as a high-potential isolated strain was generally considered a beneficial probiotic. This potential strain was selected for supplementation in feed for the two-spotted cricket (*G. bimaculatus*) to improve insect health and growth, enhance their gut microbiome, boost their immune system, and potentially increase survival rates. This application is a promising way to enhance the sustainability and efficiency of cricket farming. The probiotic strain *Lb. plantarum* TPL-2 could be incorporated into insect diets to positively influence the composition of gut microbiota. This can lead to a more balanced and beneficial microbial community, which is crucial for insect health and digestion. Moreover, probiotic supplementation can prevent the spread of diseases in insect populations and reduce the number of some human and cricket pathogens, such as *C. farmeri*, *Enterococcus* sp., *E. coli*, *K. oxytoca*, and *M. morganii*, which is a significant benefit in the context of large-scale insect rearing for food and feed. The results align with the previous study on the probiotic *Pediococcus pentosaceus* KVLB19-01, which increased feed efficiency, enhanced larval growth rates and survival, provided resistance to pathogens, and modulated gut microbiota in mealworms (*T. molitor*) [6]. Additionally, probiotic diets supplemented with *Lactobacillus* species (*Lb. casei* TISTR 390, *Lb. plantarum* TISTR 541, *Lb. curvatus* TISTR 938, and *Lb. acidophilus* TISTR 2365) have been shown to positively influence honey bee (*Apis mellifera* L.) colony populations by increasing the number of eggs, larvae, pupae, and adults [60].

## 5. Conclusions

In summary, the guts of the termite *T. propinquus* serve as a natural source of beneficial probiotic lactic acid bacteria for enhancing cricket health, growth, and resistance to diseases, potentially contributing to more sustainable and efficient cricket farming practices. The strain *Lb. plantarum* TPL-2 exhibited good probiotic characteristics with safe and antibacterial activity against important foodborne bacteria. The findings regarding probiotic agents can be applicable to food biotechnology and microbiology in the future.

## Figures and Tables

**Table 1:** Safety assessment of lactic acid bacteria (TPL-1 to TPL-5) based on antibiotic susceptibility, hemolysis, DNase activity, biogenic amine production, and detection of virulence factor genes (VFGs).

Test	Strains				
	TPL-1	TPL-2	TPL-3	TPL-4	TPL-5
Antibiotic susceptibility (inhibition zone diameter, mm)					
Ampicillin	S (48)	S (53)	S (33)	R (0)	S (46)
Penicillin	R (0)	R (0)	R (0)	R (0)	R (9)
Vancomycin	R (9)	R (0)	S (34)	R (0)	S (26)
Kanamycin	R (10)	R (15)	R (0)	R (0)	R (0)
Streptomycin	R (8)	R (11)	R (8)	R (0)	I (19)
Tetracycline	I (16)	S (24)	I (20)	I (16)	S (23)
Chloramphenicol	S (49)	S (53)	S (39)	S (29)	S (33)
Ciprofloxacin	R (0)	R (13)	S (34)	R (0)	S (24)
Erythromycin	I (19)	S (22)	S (26)	R (0)	R (12)
Hemolytic activity	Gamma	Gamma	Alpha	Alpha	Alpha
DNase activity	-	-	-	-	-
Biogenic amine production					
Histamine	+	-	-	-	+
Tyrosine	-	-	-	-	-
Putrescine	+	-	-	-	-
Cadaverine	-	-	-	-	+
Virulence factor genes (VFGs)					
*ace* (collagen-binding protein)	-	-	+	-	-
*agg* (aggregation substance)	-	-	-	-	-
*asa*1 (aggregation substance)	-	-	-	-	+
*cpd* (sex pheromone peptides)	+	-	-	-	-
*cyl*A (cytolysin)	+	-	-	-	-
*cyl*B (cytolysin)	-	-	-	-	-
*efa*Afs (cell wall adhesins)	-	-	-	-	-
*gel*E (gelatinase)	-	-	+	-	-

Data are the mean (*n* = 3); the antibiotic susceptibility test was measured breakpoints followed by standard provided by the Clinical and Laboratory Standard Institute (CLSI) guidelines: S = sensitive (≥21 mm), I = intermediate (16–20 mm), and R = resistant (≤15 mm); - = negative result, and + = positive result.

**Table 2:** Proximate analysis of crickets (*Gryllus bimaculatus*) treated with *Lactiplantibacillus plantarum* TPL-2 at 7 and 14 days of treatment.

Items (g/100 g Dry Weight)	Control		*Lb. plantarum* TPL-2	
	7 Days	14 Days	7 Days	14 Days
Ash	4.15 ± 0.01 ^a^	4.03 ± 0.09 ^a^	4.42 ± 0.01 ^b^	4.44 ± 0.01 ^b^
Crude protein	53.89 ± 0.13 ^b^	53.21 ± 0.21 ^a^	59.07 ± 0.08 ^d^	54.67 ± 0.15 ^c^
Crude fat	20.82 ± 0.16 ^b^	20.45 ± 0.15 ^b^	17.20 ± 0.18 ^a^	21.52 ± 0.28 ^c^
Crude fiber	7.44 ± 0.05 ^a^	8.42 ± 0.10 ^b^	8.05 ± 0.22 ^b^	8.22 ± 0.35 ^b^

Values were expressed as mean ± standard deviations (S.D.) of triplicate. Means in the same row with different superscript letters are significantly different (*p* < 0.05).

## Data Availability

The original contributions presented in this study are included in the article/Supplementary Material. Further inquiries can be directed to the corresponding author.

## Supplementary Materials

The following supporting information can be downloaded at: https://www.mdpi.com/article/10.3390/microorganisms14030660/s1, Table S1: Primers, reaction conditions, and product sizes used for detection of virulence factor genes (VFGs) in this study.
