# Supplementary material for: Improving Gut Microbiota and Growth Performance of Edible Crickets (Gryllus bimaculatus) by the Probiotic Lactiplantibacillus plantarum TPL-2 from the Guts of the Termite, Termes propinquus
Source: Microorganisms. 2026 Mar 14;14(3):660. doi: 10.3390/microorganisms14030660 (PMC13029225; doi:10.3390/microorganisms14030660)
Supplement: Supplementary file 1 [file microorganisms-14-00660-s001.zip › microorganisms-4177645-supplementary.pdf]

Supplementary data

Table S1. Primers, reaction conditions, and product sizes used for detection of virulence factor genes (VFGs) in this study

Gene	Sequences (5′ – 3′)	Tm (°C)	Product size (bp)
ace	Forward : CAGGCCAACATCAAGCAACA<br>Reverse : GCTTGCCTCGCCTTCTACAA	65	125
agg	Forward : AAGAAAAAGAAGTAGACCAAC<br>Reverse : AAACGGCAAGACAAGTAAATA	53	1,553
*asa*1	Forward : GCACGCTATTACGAACTATGA<br>Reverse : TAAGAAAGAACATCACCACGA	56	375
cpd	Forward : TGGTGGGTTATTTTTCAATTC<br>Reverse : TACGGCTCTGGCTTACTA	50	782
*cyl*A	Forward : ACTCGGGGATTGATAGGC<br>Reverse : GCTGCTAAAGCTGCGCTT	60	688
*cyl*B	Forward : ATTCCTACCTATGTTCTGTTA<br>Reverse : AATAAACTCTTCTTTTCCAAC	56	843
efaAfs	Forward : GACAGACCCTCACGAATA<br>Reverse : AGTTCATCATGCTGTAGTA	56	705
*gel*E	Forward : CGAAGTTGGAAAAGGAGGC<br>Reverse : GGTGAAGAAGTTACTCTGA	50	372
